# Exploring musculoskeletal injuries in the podiatry profession: an international cross sectional study

**DOI:** 10.1186/s13047-016-0185-y

**Published:** 2017-01-11

**Authors:** Cylie M. Williams, Stefania Penkala, Peter Smith, Terry Haines, Kelly-Ann Bowles

**Affiliations:** 1 Department of Physiotherapy, Monash University, Frankston, VIC 3199 Australia; 2 University of Western Sydney, School of Science and Health, Penrith, NSW 1797 Australia; 3 Monash University, Epidemiology and Preventative Medicine, Prahran, VIC 3181 Australia; 4 Institute for Work & Health, Toronto, ON Canada; 5 Dalla Lana School of Public Health, University of Toronto, Toronto, Canada; 6 Monash Health, Allied Health Research Unit, Cheltenham, VIC 3192 Australia; 7 Department of Community Emergency Health and Paramedic Practice, Monash University, Melbourne, VIC 3199 Australia

**Keywords:** Health workforce, Ergonomics, Back pain, Musculoskeletal disorders

## Abstract

**Background:**

Workplace injury is an international costly burden. Health care workers are an essential component to managing musculoskeletal disorders, however in doing this, they may increase their own susceptibility. While there is substantial evidence about work-related musculoskeletal disorders across the health workforce, understanding risk factors in specific occupational groups, such as podiatry, is limited.

The primary aim of this study was to determine the prevalence and intensity of work related low back pain in podiatrists.

**Methods:**

This was an international cross-sectional survey targeting podiatrists in Australia, New Zealand and the United Kingdom. The survey had two components; general demographic variables and variables relating to general musculoskeletal pain in general or podiatry work-related musculoskeletal pain. Multivariable regression analyses were used to identify factors associated with musculoskeletal stiffness and pain and low back pain intensity. Thematic analysis was used to group comments podiatrists made about their musculoskeletal health.

**Results:**

There were 948 survey responses (5% of Australian, New Zealand and United Kingdom registered podiatrists). There were 719 (76%) podiatrists reporting musculoskeletal pain as a result of their work practices throughout their career. The majority of injuries reported were in the first five years of practice (*n* = 320, 45%). The body area reported as being the location of the most significant injury was the low back (203 of 705 responses, 29%). Being female (*p* < 0.001) and working in private practice (*p* = 0.003) was associated with musculoskeletal pain or stiffness in the past 12 months. There were no variables associated with pain or stiffness in the past four weeks. Being female was the only variable associated with higher pain (*p* = 0.018). There were four main themes to workplace musculoskeletal pain: 1. Organisational and procedural responses to injury, 2. Giving up work, taking time off, reducing hours, 3. Maintaining good musculoskeletal health and 4. Environmental change.

**Conclusions:**

The postures that podiatrists hold while treating patients appear to impact on musculoskeletal pain and stiffness. Recently graduated and female podiatrists are at higher risk of injury. There is a need for the profession to consider how they move and take care of their own musculoskeletal health.

**Electronic supplementary material:**

The online version of this article (doi:10.1186/s13047-016-0185-y) contains supplementary material, which is available to authorized users.

## Background

Workplace injury is a costly burden for both employers and employees internationally. In the United Kingdom the cost of workplace injury was estimated to be £4.9 billion in 2013/14 [1] with musculoskeletal disorders accounting for 44% of all workplace illness in this country [2]. These figures are even higher in Australia where musculoskeletal disorders account for 60% of serious workplace compensation claims [3]. Musculoskeletal disorders are one of the most expensive workplace illnesses to manage [4]. Compensation costs for work-related musculoskeletal disorders in New Zealand accounted for almost NZD$150 million between 2009 and 2010 [5], excluding the indirect costs to individuals and workplace productivity. As musculoskeletal disorders become increasingly common in the workplace [6], workplace strategic recommendations prioritise addressing these [7].

Health care workers are an essential component to managing musculoskeletal disorders, however in doing this, they may increase their own susceptibility. Workers in health and community services reported higher levels of workplace injuries than the Australian average with 77 injuries per 1000 worker in health compared to 69 injures per 1000 workers across all industries [8]. Burnout, high staff turnover rates, and poor job satisfaction have been attributed to health care workforce shortages and musculoskeletal disorders [9–11]. Most research has investigated work-related musculoskeletal disorders in nursing, with an emphasis on low back pain attributed to strenuous patient handling activities such as lifting [12]. Shoulder and neck pain in nursing and neck and wrist pain in dentistry [13] have also received attention in the literature.

While there is substantial evidence about work-related musculoskeletal disorders across the health workforce, understanding risk factors in specific occupational groups, such podiatry, is limited. Podiatrists have ergonomic challenges that may increase their risk of musculoskeletal pain such as working in sustained and awkward postures, performing repetitive manual skills requiring high precision, while dealing with equipment related forces and vibration [6, 14–16]. The point prevalence of work-related musculoskeletal disorders in podiatry has previously been reported to be between 66 and 88% in the UK [17] and Australia [18] in samples between 32 and 347 podiatrists. In general, the expected work-related musculoskeletal disorders in podiatry practice is reported to be above the average compared to other health professionals [19].

Given the greater impact of low back problems on daily activities [18] the primary aim of this study was to describe the prevalence and intensity of work related low back pain in podiatrists. The secondary aims were to explore the factors associated with restriction in activity.

## Method

### Study Design

This study was an international cross-sectional survey targeting podiatrists in Australia, New Zealand and the United Kingdom. The Monash University Human Research Ethics Committee, Victoria, Australia, approved this study. (MUHREC approval – CF16/1009 – 2,016,000,538).

### Participants and setting

Eligible participants were all registered podiatrists within Australia (*n* = 4,626) [20], New Zealand (*n* = 382) [21] and the United Kingdom (*n* = 13,111) [22]. The survey was advertised in each country at local podiatry conferences and seminars, disseminated by email flyers, newsletters and online media (Facebook, LinkedIn and Twitter), through the Australian Podiatry Council, state based Australian Podiatry Associations, Podiatry NZ, and The Society of Chiropodists & Podiatrists.

### Measurements

All participant data were collected via a custom-developed, self-report, online survey (Appendix 1). The survey had two components; general demographic variables and variables relating to general musculoskeletal pain in general or podiatry work-related musculoskeletal pain. General demographic variables included gender, age group, country, region of practice, recency of practice and primary work roles. Participants were also requested to identify the percentage of time they worked in each of the following workplaces: private practice, public sector (community), public sector (hospital), and non-clinical (including education, research or administrative settings).

Questions about back pain were based on a previous study which developed a consensus based back pain definition [23]. Additional questions added were based on previously published work [24] focusing on musculoskeletal disorders in physiotherapists. These questions asked podiatrists to recall over the past 12 months, the number of occasions that work related pain or injury that;

1. Lasted longer than seven days
2. Prevented working for more than a day
3. Affected activities of daily living
4. Resulted in a health professional consultation

Podiatrists were also asked to identify the body region where the most severe work- related pain was experienced, if there was a history of work related injury specific to their pain throughout their career, and the time-frame in which the injury occurred in their career.

Any podiatrists who reported any pain during their career were then asked how they dealt with work related musculoskeletal pain and if they reported this to their management. Podiatrists were asked if they had musculoskeletal pain or stiffness specifically related to their low back in the past 4 weeks and to quantify any pain experienced in the previous four weeks on an eleven-point visual analogue scale (VAS) where 0 means “no pain” and 10 means the “worst pain imaginable”. A free text box at the end of the survey prompted further information from participants by asking “If you have any comments about the questions or your responses within this survey, please add within the text box below”. To reduce missing data, a forced response was used throughout the survey and the “don’t know” option was removed.

### Procedure

Following dissemination of the survey link, each participant gave consent and completed the survey online. The survey was open from the 13^th^ of April, 2016 to the 28^th^ of July, 2016. There was regular advertising of the survey via the same modalities as outlined in the original survey dissemination above and all podiatrists were encouraged to share the advertisement with fellow podiatrists via their networks.

The responses were collected using SurveyMonkey® online survey software (Additional file 1) [25] and utilized skip logic when podiatrists indicated no musculoskeletal pain or stiffness relating to their job role. The survey was set to ensure internal fidelity of each section with the participant not able to continue the survey without full completion of the previous section. The participants were able to withdraw from the survey at any time by closing the browser and any non-completion was treated as missing data for the remaining non-completed variables.

### Analysis

Data were analysed using Stata 13 [26]. Descriptive statistics were used to report on the distribution of each variable. Univariate regression analyses were used to identify candidate variables for inclusion in the definitive multivariable model. Only variables that had a univariate association with the dependent variable were considered where the *p*-value was 0.10 or less for inclusion in the preliminary multivariable model. This preliminary multivariable model was then reduced by removing variables one-at-a-time based on the variable with the highest adjusted *p*-value. This continued until all variables remaining in the multivariable model had an adjusted *p*-value of less than 0.05. This final model was referred to as the definitive multivariable model. Only data was used data from respondents where all questions had been answered (complete case analysis). Multivariable regression analyses were used to identify factors independently associated with the dichotomous response of musculoskeletal pain or stiffness within the past 12 months, low back pain within the past four weeks and and continuous outcome ranging from 0 to 10 for low back pain intensity, where 10 equals maximum pain. For participants who had no pain and skipped this question, their response was allocated 0 during analysis of continuous outcomes. The model predictor variables included gender, age, percentage of time at private practice, public sector community health, public sector acute and non-clinical, the recency of practice and workload.

Inductive thematic analysis of the open text questions was undertaken by hand. Inductive thematic analysis allows for the content of statements to be analysed in full and where concepts or categories can be derived from the data in an inductive manner [27]. In this analysis, themes were generated from the statements as opposed to themes being developed by the investigators [27]. The full statements were manually grouped against meaningful concepts. These concepts were reviewed with themes developed. Even if a theme was addressed in one sentence, the statement was included under that theme. The analysis also took an iterative approach where as new themes were developed, earlier statements were recoded.

## Results

Following removal of consent only responses, there were 948 survey responses (5% of Australian, New Zealand and United Kingdom registered podiatrists). Table 1 displays a breakdown of the demographics of participants taking into account missing data from non-completion due to early exit from the survey. Table 1 also gives the demographic breakdown based on individual country responses, Australia (*n* = 652, 14% responses from 4626 podiatrists), New Zealand (*n* = 40, 10% of 382 registered podiatrists), United Kingdom (*n* = 256, 2% of 13,111 registered podiatrists).

**Table 1:** Demographics of participants *n* (%)

	Total responses *n* = *948*	Australia *n* = 652, 69% of total responses	New Zealand *n* = 40, 4% of total responses	United Kingdom *N* = 256, 27% of total responses
	*n* (%) or Mean (SD)	*n* (%) or Mean (SD)	*n* (%) or Mean (SD)	*n* (%) or Mean (SD)
Gender	*n* (%)	*n* (%)	*n* (%)	*n* (%)
Male	275 (29)	198 (30)	6 (15)	71 (28)
Female	670 (71)	451 (69)	34 (85)	185 (72)
Prefer not to answer	1 (0)	1 (0)	0 (0)	0 (0)
Intersex	2 (0)	2 (0)	0 (0)	0 (0)
Age group	*n* (%)	*n* (%)	*n* (%)	*n* (%)
Under 25	65 (7)	48 (7)	6 (15)	11 (4)
25 - 29	188 (20)	156 (24)	6 (15)	26 (10)
30 – 34	145 (15)	111 (72)	7 (18)	27 (11)
35 – 39	119 (13)	78 (12)	3 (8)	38 (15)
40 – 44	126 (13)	72 (11)	6 (15)	48 (19)
45 – 49	115 (12)	72 (11)	2 (5)	41 (16)
50 – 54	107 (11)	65 (10)	7 (18)	35 (14)
55 – 59	61 (6)	37 (6)	2 (5)	22 (9)
60 – 64	17 (2)	11 (2)	1 (3)	5 (2)
65 – 79	4 (0)	2 (0)	0 (0)	2 (1)
80+	1 (0)	0 (0)	0 (0)	0 (0)
Workload	*n* (%)	*n* (%)	*n* (%)	*n* (%)
Full time	554 (59)	381 (58)	22 (55)	151 (59)
3-4 days	276 (42)	178 (27)	13 (33)	85 (33)
2 days or less	105 (16)	83 (12)	5 (13)	17 (7)
Do not currently practice as a podiatrist	12 (1)	10 (2)	0 (0)	2 (1)
Working environment	Mean % of time	Mean % of time (SD)	Mean % of time (SD)	Mean % of time (SD)
Public sector (hospital)	(SD)	45 (40)	8 (24)	21 (28)
Public sector (community)	34 (38)	54 (36)	26 (29)	72 (33)
Private Practice	60 (37)	82 (29)	80 (24)	68 (38)
Non-clinical	78 (31)	33 (30)	14 (16)	22 (21)
Do not currently practice as a podiatrist	29 (28) 16 (37)	20 (41)	0 (0)	13 (34)
Years of practice	*n* (%)	*n* (%)	*n* (%)	*n* (%)
0–5	242 (26)	178 (27)	10 (25)	54 (21)
6–10	202 (21)	149 (23)	11 (28)	42 (16)
11–15	148 (16)	93 (14)	6 (15)	49 (10)
15+	355 (37)	232 (36)	13 (33)	110 (43)
Primary work role	Total responses=	Total responses = 824	Total responses =52	Total responses: 324
Patient/Client podiatry service provision	1198	595 (72)	38 (73)	239 (74)
Supervision or mentor of other podiatrists	874 (73)	87 (11)	6 (12)	35 (11)
Manager/Team leader of other podiatrists	125 (10)	84 (10)	4 (8)	30 (9)
Administration only within podiatry (includes research/education)	120 (10) 66 (6)	43 (5)	4 (8)	15 (5)
Do not currently practice as a podiatrist		5 (1)	0	2 (1)
Other	8 (1) 5 (0)	10 (1)	0	3 (1)

**Fig. 1:**
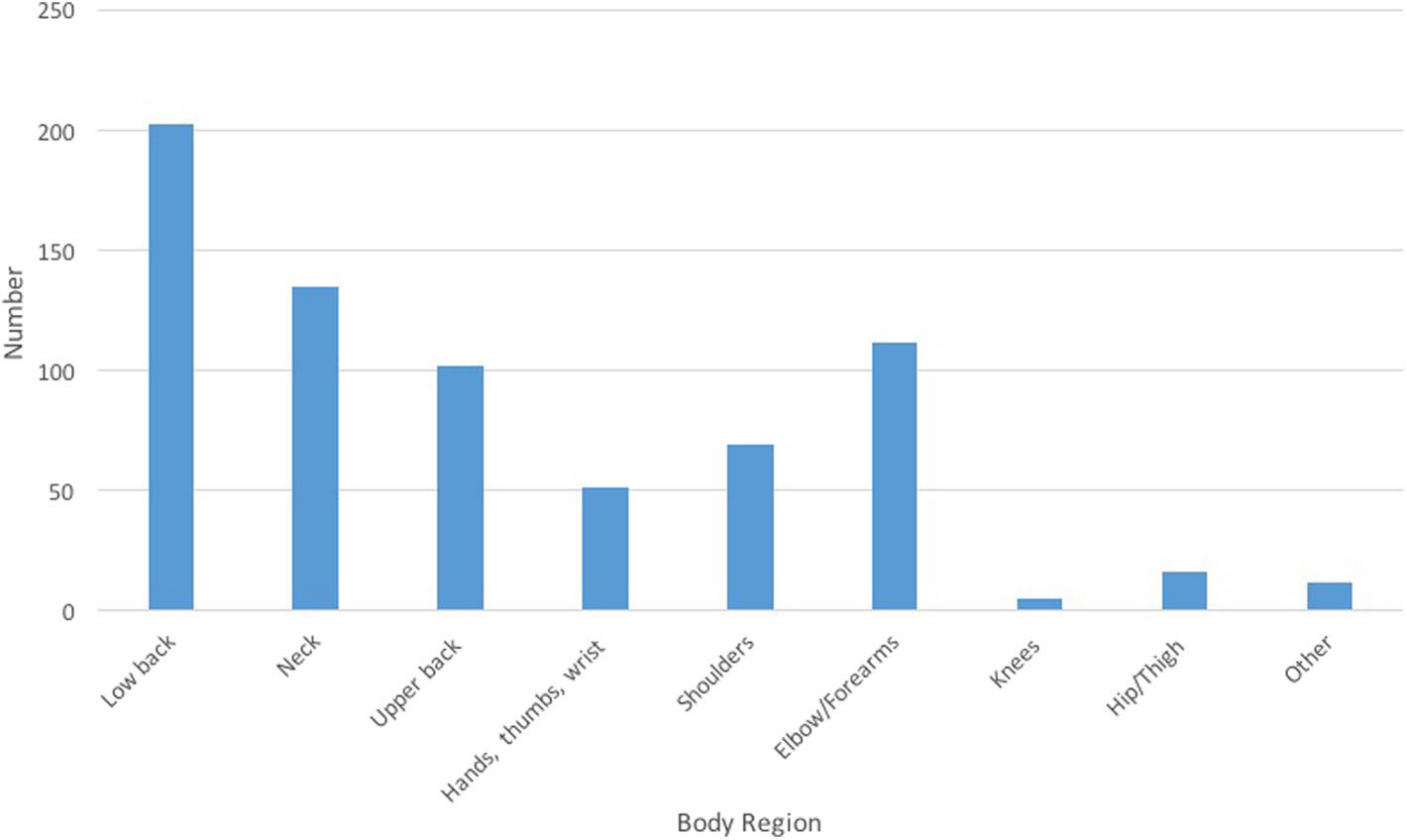
Body Region of most significant injury (*n* = 705)

Overall from the 948 podiatrists who responded, 719 (76%) reported musculoskeletal pain as a result of their work practices throughout their career. Of the 705 responses that indicated the area of most significant injury, 29% reported the low back as the area of most significant injury (*n* = 203). Table 2 provides a breakdown of the body region of most significant injury (also found in Fig. 1), age at the time of injury, stage in career, changes in practice and if the problem was reported to a manager or supervisor. Of the total number of significant injuries, 533 (75%) changed their practice as a result of the injury however only 408 (58%) reported this injury to a supervisor or manager.

**Table 2:** Body Region of most significant injury throughout career

	Distribution of injuries across body	Age at Injury^a^					Career stage of Injury^b^					Change practice^b^		Reported injury to management**	
		Under 25	25-34	35-44	45-54	55-64	During training	First 5 years of practice	6-10 years of practice	11 to 15 years of practice	15+ after graduating	Yes	No	Yes	No
All regions (*n*, %)	705 (100%)	131 (1%)	313 (44%)	139 (2%)	98 (14%)	24 (3%)	12 (2%)	320 (45%)	179 (25%)	70 (10%)	119 (17%)	533 (75%)	171 (24%)	274 (39%)	408 (58%)
Low back	203 (29%)	44 (22%)	97 (48%)	45 (22%)	14 (7%)	3 (1%)	5 (2%)	107 (53%)	46 (23%)	17 (8%)	26 (13%)	168 (83%)	35 (17%)	84 (41%)	111 (55%)
Neck	135 (19%)	18 (13%)	66 (49%)	25 (19%)	18 (13%)	8 (6%)	1 (1%)	54 (40%)	41 (30%)	20 (15%)	19 (14%)	102 (76%)	33 (24%)	50 (37%)	82 (61%)
Upper back	102 (15%)	22 (21%)	53 (52%)	14 (14%)	12 (12%)	1 (1%)	1 (1%)	50 (48%)	31 (30%)	6 (7%)	13 (13%)	73 (72%)	29 (28%)	26 (25%)	76 (75%)
Hands, thumbs, wrist	51 (7%)	5 (10%)	17 (33%)	10 (20%)	13 (25%)	6 (12%)	1 (2%)	18 (35%)	8 (17%)	7 (14%)	17 (33%)	34 (67%)	17 (33%)	18 (35%)	29 (57%)
Shoulders	69 (10%)	9 (13%)	30 (45%)	15 (22%)	13 (19%)	2 (3%)	1 (1%)	28 (41%)	20 (29%)	6 (9%)	13 (19%)	49 (71%)	20 (29%)	24 (35%)	42 (61%)
Elbow/Forearms	112 (16%)	30 (27%)	40 (36%)	20 (18%)	19 (17%)	3 (3%)	3 (3%)	53 (47%)	24 (21%)	8 (7%)	23 (21%)	80 (71%)	32 (29%)	60 (53%)	49 (44%)
Knees	5 (1%)	0 (0%)	1 (20%)	3 (60%)	1 (20%)	0 (0%)	0 (0%)	2 (40%)	2 (40%)	1 (20%)	0 (0%)	5 (100%)	0 (0%)	3 (60%)	2 (40%)
Hip/Thigh	16 (2%)	1 (6%)	5 (31%)	4 (25%)	5 (31%)	1 (6%)	0 (0%)	4 (25%)	4 (25%)	2 (12%)	6 (38%)	14 (88%)	2 (12%)	5 (31%)	10 (63%)
Other	12 (1%)	2 (17%)	4 (33%)	3 (25%)	3 (25%)	0 (0%)	0 (0%)	4 (33%)	3 (25%)	3 (25%)	2 (17%)	8 (67%)	3 (25%)	4 (33%)	7 (58%)

^a^No injuries reported over 64 years

^b^Missing data responses from drop outs

Of the problems experienced by podiatrists over the last 12 months lasting longer than 7 days, more occasions of musculoskeletal pain or stiffness, of either single or constant episodes, occurred in the shoulders (Median = 1, IQR 0,5), neck (Median = 2, IQR = 1,6) and upper back (Median = 1, IQR = 0,5) and low back (Median = 2, IQR 1,5) (Table 3). There were very few occurrences where a podiatrist took more than a day off for musculoskeletal pain in the past twelve months. Occasions of pain or stiffness at the low back impacted activities of daily living more than any other body part (Median = 1, IQR = 0,2) (Table 3). Only one participant reported low back pain constantly impacted activities of daily living, all other participants reported a range of occasions between 0–25 in the past 12 months. Podiatrists regularly sought support from health care professionals (Table 3). Podiatrists presented for health professional support more frequently for pain or stiffness at the neck (Median = 2, IQR = 1,4), low back (Median = 1, IQR = 1,3) or upper back pain (Median = 1, IQR = 0,4) than any other body part. It is unknown if these appointments were in clusters (i.e. seeing a clinician every week day for a time period of acute pain) or if regular bi-weekly, weekly or monthly appointments.

**Table 3:** Impact and results of occasions of pain at body regions

	Lasted longer than 7 days		Prevented work for >1 day		Affecting ADLs		Saw health professional	
	Median, (IQR)	N, Range	Median, (IQR)	N, Range	Median, (IQR)	N, Range	Median, (IQR)	N, Range
Low back	2 (1,5)	400, 0-365	0, (0,1)	243, 0-30	1, (0,2)	251, 0-365	1, (1,3)	346, 0-150
Neck	2 (1,6)	404, 0-365	0, (0,1)	219, 0-50	0, (0,1)	225, 0-30	2, (1,4)	337, 0-150
Upper back	1, (0,5)	296, 0-365	0, (0,0)	168, 0-30	0, (0,1)	183, 0-100	1, (0,4)	265, 0-60
Hands, thumbs, wrist	1, (1,5)	339, 0-365	0, (0,0)	178, 0-30	0,(0–1)	186, 0-100	0, (0,2)	220, 0-30
Shoulders	1 (1,5)	270, 0-365	0, (0,0)	163, 0-100	0, (0,1)	170, 0-100	1 (0,3)	230, 0-100
Elbow/Forearms	1, (0,3)	206, 0-365	0, (0,0)	149, 0-15	0, (0,0)	150, 0-20	0, (0,1)	177, 0-50
Knees	0, (0–1)	159, 0-365	0, (0,0)	142, 0-42	0, (0,0)	136, 0-42	0, (0,0)	138, 0-150
Hip/Thigh	0, (0–2)	175, 0-365	0, (0,0)	151, 0-5	0, (0,0)	147, 0-18	0, (0,1)	160, 0-150
Feet/ankles	0, (0,1)	148, 0-365	0, (0,0)	143, 0-21	0, (0,0)	136, 0-21	0, (0,0)	132, 0-15

Of the 719 podiatrists reporting work-related musculoskeletal pain during their career, 427 (59%) reported low back pain in the past four weeks with a mean (SD) visual analogue score of 3.8 (1.9) on a scale of 0 to 10. Being female (*p* < 0.001) and working in private practice (*p* = 0.003) was associated with musculoskeletal pain or stiffness in the past 12 months (Table 4). There were no variables associated with pain or stiffness in the past four weeks. Being female was the only variable associated with higher pain (*p* = 0.018) (Table 4).

**Table 4:** Variables associated with greater frequencies or intensity of pain or stiffness over time determined through multivariable analysis

	*n* (%)	Gender (Female) Odds Ratio (95% CI), p	Private Practice Odds Ratio (95% CI), p
Musculoskeletal stiffness, pain or injury in past 12 months	653 of 901 responses (72%)	2.16 [1.52, 3.08], <0.001	1.01 [1.00, 1.01], 0.003
Low back pain or stiffness in past 4 weeks^a^	427 of 865 responses (49%)^a^		
Low back pain as measured with the VAS^b^	948 of 948 responses (100%)^a^	1.48 [1.07, 2.01], 0.018	

^a^No variables associated

^b^Where no musculoskeletal pain was recorded from podiatrists, a 0 was allocated within analysis

There were four main themes identified from the 99 comments from podiatrists at the end of the survey.

1. Organisational and procedural responses to injury
  There were few positive comments in relation to the impact and responses of employers and how they deal with work health and safety issues raised by employees. Responses such as “I’ve found working for a small business often means less OHS [Occupational Health and Safety] *awareness for staff*, *compared to the hospital system*” (*29*) and “*My neck problems never materialised before I started working as a podiatrist* \*and the NHS* [National Health Service] *Occupational Health were quick to inform me that it was not as a result of work related problems*” (*35*), highlighted these challenges. Podiatrists also reported inaction by management, saying “[*I*] *Raised concerns with management when floor issue was initially noted*, *but no action. Proceeded to complete incident form when back pain occurred* – *currently waiting on ergonomic assessment and not working at that site in the meantime*” (*18*).
2. Giving up work, taking time off, reducing hours
  There were numerous comments about how podiatrists reduced hours and/or changed client types to manage the impact of musculoskeletal pain, including giving up work in the profession altogether. These comments appeared to greatly impact health related quality of life as a result of constant pain, and the need to pursue other avenues of income. One podiatrist reported “*My pains have led me having to stop practicing. It has been 8 years and am relying on long term pain relief*/*pain management. Had several referrals to rheumatologists*, *physios and been told I just have to live with it*” (*38*), and another, “*I am at 6.5 years* [recency of practice] *and 28 years old and I don*’*t see myself being able to sustain this* ‘*constant*’ *podiatry general treatments for much longer* (*maybe another 5 years or so*)” (*79*). It appeared that this was a challenge right from being a newly qualified podiatrists with statements such as “*As a new grad you did whatever work you were asked to do*, *and often worked in much busier clinics* […] *An older*, *experienced podiatrist is more likely to work with more complex clients and therefore more time to treat less and less patients per day*” (*62*). Podiatrists who worked in private practice also comment about the nature of their work making it extremely difficult to take time off. “*When you*’*re self*-*employed you can*’*t take time off even if in pain*” (*32*).
3. Maintaining good musculoskeletal health
  Many podiatrists were reflective on what they wish they knew as a new graduate and what they were doing to maintain and minimise the impact of the job on their bodies. One podiatrist stated “*I became a moving and handling advisor due to many musculoskeletal* (*MSK*) *disorders related to podiatry* […] *With my new role I provide advice to podiatry staff re ways to reduce MSK* [musculoskeletal] *disorders*” (*34*). Another reflected that “*I wish I had known the shocking impact of Podiatry on my upper back and neck* […] *People need to be aware of these factors before working as a podiatrist*” (*27*).
  There were concerns that constant pain was difficult to manage and a frustration of being reliant on pharmaceuticals, surgery, or other direct intervention as a result of their chosen profession. “*I live with constant neck and back pain. It*’*s become part of everyday life as a podiatrist*” (*89*), “*My thumb*, *wrist and forearm injuries are caused by two incidents involving patient care that I am awaiting surgery to resolve*” (*42*) and “*My back pain got so bad I was seeing a chiro* [chiropractor] *one week*, *the physio the next*, *then the sport massage the following week*” (*85*) were all examples of self -management strategies.
4. Environmental changes
  Podiatrists reported using their office equipment such as specific seats, made regular postural adjustments while treatment and participated in regular exercise as a way to minimise musculoskeletal pain. Interestingly, there were a number of comments regarding the use of practitioners seating that is common to the profession, for example “*I stopped using the saddle chair and my symptoms of pain settled and the sharp tendinitis morning pain was eliminated within 3 months. I still try and sit and stand 50*/*50*, *however I am quite certain the saddle chair was the reason for my shoulder pain*” (*79*). Proactive self-care strategies included “*Going to the gym and becoming very fit*, *working on my core strength has made a huge difference to back problems I*’*ve had in the past*” (*74*).

## Discussion

The aim of this project was to establish a prevalence of musculoskeletal injuries amongst podiatrist in Australia, New Zealand and the UK. This research also aimed to identify which body sites were most problematic for podiatrist and had a greater effect on the professional practice and life outside of work. These results indicate that 76% of podiatrists had experienced musculoskeletal pain attributed to work during their career, with pain and/or injury to the low back the most common site to negatively affect work and outside life.

Similar small studies of the podiatry profession in the UK, Australia and Spain found up to 20% of musculoskeletal injuries within a 12 month period specific to the low back, neck, upper back, wrist and hands [18, 28, 29]. While low back problems in these studies were only around half the prevalence in dental and nursing practice [12, 30], two other podiatry studies [28, 19] reported a higher prevalence of low back problems in a one to seven day capture period. Importantly, multiple sites of work-related musculoskeletal injuries are reported at the one time [17, 18]. The last survey of podiatrists in Australia, found 88% (*n* = 304) of podiatrists reporting a musculoskeletal injuries within the last 12 months, and that on average three areas of the body were affected at the same time [18].

Similar to the dental profession, females reported more frequent and a higher intensity of pain [31]. This may be due to the similarities in sitting postures between the professions as the same was not found in the physiotherapy profession [32]. In population studies, older females have also reported more frequent or more intense back pain [33]. It is possible that this finding in podiatry is representative of the population as a whole.

Other studies have reported seeking treatment for neck pain was the most common site, followed by the shoulders, upper and low back [18]. In contrast to previous work [18], this survey of podiatrists determined that low back problems were more likely to affect daily activities followed by the neck, wrists and hands, shoulders and upper back . It is possible that there is a level of under-reporting to management or over-reporting in surveys due to response bias. This present research also did not ask podiatrists to identify if they worked as sole practitioners, therefore the under reporting of injuries may simply be a result of having no manager to report to. A previous study reporting interviews with seven podiatrists indicated podiatrists had a tendency to persevere with musculoskeletal disorders and associated pain and dysfunction in the hands rather than taking time off ‘*as you don*’*t want to let anyone down*’ [16].

Recently graduated podiatrists may work in postures that result in increased susceptibility to musculoskeletal injury. Forty-five percent of the respondents experienced the most serious injury in the five years post training. Participants reported that the most frequent time-frame where injury occurred, regardless of which body site, was the first five years of working. This research is consistent with previous results from other health disciplines, with Glover et al. [24] identifying five years post-graduation as the most at risk time-point to experience a serious musculoskeletal injury.

Previous research has proposed that the true impact of musculoskeletal injury amongst health care professionals is hidden as result of the tendency to informally seek treatment from a colleague, and as many as 1 in 6 leave the profession due to injury [34]. The results have identified that 75% of respondents with an injury did have to change the way they practised and 58% did not report the injury to a manager. These results support anecdotal belief that health professionals do not report musculoskeletal pain and injuries to managers as they do not want this to affect career prospects. It should be noted that some health professionals do work as private consultants and do not have a manager to report to, however these results did show that reporting of injuries to available managers was still low. As it is suggested that it costs approximately $40,000 to train a podiatrist in Australia [35, 36, 37], the profession needs to ensure that mechanisms are put into place so that health professionals can enjoy their career to their full potential. This could include identifying the risk factors that can lead to a musculoskeletal injury, and ensuring that support is available for injured workers.

This was the largest self report of musculoskeletal disorders in the podiatry profession however the sample size is still a limitation of the study and its generalizability to the rest of the profession. This sample size represents 5% of the podiatry profession across Australia, New Zealand and the United Kingdom. While this is a low response rate, a reasonable variation in the sample was observed across the different outcomes and predictor variables. As such, while this limits in the ability to accurately describe the true prevalence of pain in the podiatry profession as a whole, there is still the ability to examine the relationships between variables, such as the distribution of pain across body parts, or the predictors of different pain outcomes, as these estimates do not necessarily require representative samples [37, 38]. There was potential for an over reporting of pain due to self selection bias. It is postulated that podiatrists who had pain were more likely to respond to the survey due to an eagerness to share their experience. This has some potential to limit the generalizability of the results to the whole profession however the results are in line with other similar studies. Longitudinal studies of podiatrists throughout their career are needed to better understand the development of any musculoskeletal impacts. Real time monitoring of postures may also improve understanding of the mechanism of pain or stiffness. Universities should also consider how treating postures are taught to student podiatrists and embed good postural habits at the beginning of their career.

## Conclusion

The postures that podiatrists hold while treating patients may impact on musculoskeletal pain and stiffness. This pain and stiffness can impact on activities of daily living and cause podiatrists to change work practices. Podiatrists reported more injuries at the beginning of the careers. Universities training podiatrists and employers of recently graduated podiatrist should especially be aware of treatment environments and case-loads that may predispose podiatrists to more injuries. There is a need for the profession to consider how they move and take care of their own musculoskeletal health.

## Additional file

Additional file 1: FLEXAR survey consent and questions. (PDF 434 kb)
