# Supplementary material for: Exploring musculoskeletal injuries in the podiatry profession: an international cross sectional study
Source: J Foot Ankle Res. 2017 Jan 11;10:3. doi: 10.1186/s13047-016-0185-y (PMC5225550; doi:10.1186/s13047-016-0185-y)
Supplement: Additional file 1: — FLEXAR survey consent and questions. (PDF 434 kb) [file 13047_2016_185_MOESM1_ESM.pdf]

Additional file 1. FLEXAR survey consent and questions

* 1. FLEXAR FOR PODIATRISTS STUDY
Project number: CF16/1009 - 2016000538

Our names are Kelly-Ann Bowles, Cylie Williams, Stefania Penkala, Peter Smith, Ross Iles, and Terry Haines. We hold research positions at Monash University and the Western Sydney University, Australia.

You are invited to take part in the FLEXAR for Podiatrists study. Please read this explanatory statement in full before making a decision.

We are interested in understanding the prevalence of musculoskeletal injuries in Podiatrists who work in Australia, New Zealand and the United Kingdom.

You are invited to take part in this research if you are a qualified and registered podiatrist. We are interested in hearing from podiatrists who work in any or all of the different sectors: private (private practices), public (hospitals, community health services, NGO's, NHS) and/or tertiary institutions (Universities, colleges). This research has been approved by the Research Ethics Committee of Monash University, Victoria, Australia.

It is not expected that you will directly benefit from this research and there is no payment for being part of this research. It is hoped that by conducting this research, a better understanding is gained about the musculoskeletal impact of the international podiatry profession. If you choose to participate in this survey, it will take approximately 10 minutes. As this is an online survey, you can complete this in your own time. There are no questions in this survey that are expected to cause emotional distress or discomfort.

You can withdraw from this survey at any time by closing your Internet browser window. Being part of this study is voluntary and you are under no obligation to consent to participate. However, if you do consent and answer questions, anything you have answered may be used within the research. You will not be able to withdraw the answers to any questions you have answered.

The results of this survey are confidential and you will not be asked to give any identifying information therefore can not and/or will not be identified from your responses. Your responses will only be viewed by the research team. The responses that are collected will be stored in accordance with Australian Privacy Regulations, and will be kept as a data file that is password protected and kept for 5 years. After this, the file will be deleted. A report of this study will be submitted for publication, but individuals will not be identifiable in this report. At the completion of this research, a one page statement will be distributed to Australian Podiatry Association member organisations, Podiatry New Zealand and the Society of Chiropodists and Podiatrists for dissemination.

If you could like a summary of the finding emailed to you, you can make record your email address at the

end of the survey. This will in no way be linked to your answers and only viewed by the Chief Investigator - Kelly-Ann Bowles.

It is expected that the results will be published in a peer reviewed journal.

If you would like to contact the research team about aspects of this study or have a complaint concerning the manner in which this research is being conducted, please contact:

Kelly-Ann Bowles
kelly-ann.bowles@monash.edu

Cylie Williams
cylie.williams@monash.edu

OR

Complaints

Should you have any concerns or complaints about the conduct of the project, you are welcome to contact the Executive Officer, Monash University Human Research Ethics (MUHREC):

Executive Officer
Monash University Human Research Ethic

○ I am an Australian, New Zealand or United Kingdom Podiatrist, and consent

* 2. What is your gender?

- Male
- Female
- Intersex
- Prefer not to answer

* 3. What is your age?

- Under 25
- 25 to 29
- 30 to 34
- 35 to 39
- 40 to 44
- 45 to 49
- 50 to 54
- 55 to 59
- 60 to 64
- 65 to 69
- 70 to 74
- 75 to 79
- 80 +

* 4. Where is your principal place of practice?

- ○ Australian Capital Territory, Australia
- ○ New South Wales, Australia
- ○ Northern Territory, Australia
- ○ Queensland, Australia
- ○ South Australia, Australia
- ○ Tasmania, Australia
- ○ Victoria, Australia
- ○ Western Australia
- ○ No principle place of practice
- ○ North Island, New Zealand
- ○ South Island, New Zealand
- ○ Scotland, UK
- ○ Northern England, UK
- ○ Midlands, UK
- ○ South-East England, UK
- ○ London, UK
- ○ South-West England, UK
- ○ Wales, UK
- ○ Northern Ireland, UK
- ○ Other (please specify)

* 5. In which geographical location is your principle place of practice?

- Metropolitan
- Rural
- Remote
- Regional
- Other (please specify)

* 6. What is your current average workload practicing as a podiatrist?

- Full time
- 4 days per week
- 3 days per week
- 2 days per week
- 1 day per week
- Less frequent than 1 day per week
- Do not currently practice as a podiatrist

* 7. What is your current main working environment (must add up to 100%)?

Public sector - hospital (treating patients who are admitted as inpatients)

Public sector - community health (treating patients who come to an outpatient clinic or come from home to see you and funded through the public health or NHS system)

Private practice (Patient pays with or without private health insurance)

Non-clinical (includes administration, management and research in either public sector, private or university)

Do not currently practice as a podiatrist

* 8. How many years have you been practicing (in full time equivalent)?

- ○ 0 to 5 years
- ○ 6 to 10 years
- ○ 11 to 15 years
- ○ 15 + years

* 9. Please describe your current PRIMARY work role (you can choose up to 2)?

- ☐ Patient/Client podiatry service provision
- ☐ Supervision or mentor of other podiatrists
- ☐ Manager/Team leader of other podiatrists
- ☐ Administration only within podiatry (includes research/education)
- ☐ Do not currently practice as a podiatrist
- ☐ Other (please specify)

* 10. We are now interested in the prevalence of musculoskeletal pain or injuries that podiatrists may experience while performing their work roles. We are not interested in pain or injuries that occur as a result of sport or an accident external to your work role. For the purpose of this survey, musculoskeletal stiffness due to your work role is viewed as an injury if it results in pain or causes you to change the way you do things at work or at home.

Have you had any work related musculoskeletal pain or injuries in the last 12 months?

- Yes
- No

11. Please record the number of instances of pain or injury to each body region in the last 12 months that resulted in symptoms lasting for 7 days or longer.

Low back

Neck

Upper back

Thumbs

Shoulder

Hand/Wrist

Elbow/forearm

Knees

Hips/Thighs

Ankles/Feet

12. Please record the number of instances of pain or injury to each body region in the last 12 months that prevented you from working for more than a day.

Low back	
Neck	
Upper back	
Thumbs	
Shoulder	
Hand/Wrist	
Elbow/Forearm	
Knees	
Hip/Thigh	
Ankles/Feet	

13. Please record the number of instances of pain or injury to each body region in the last 12 months that prevented you from performing activities of daily living such as driving a car, caring for family etc.

Low back	
Neck	
Upper Back	
Thumbs	
Shoulder	
Hand/Wrist	
Elbow/Forearm	
Knees	
Hip/Thigh	
Ankles/Feet	

14. Please record the number of instances of pain or injury for each body region over the last 12 months that resulted in you seeing a health professional (Doctor, physiotherapist etc)

Low Back	
Neck	
Upper Back	
Thumbs	
Shoulder	
Wrist/Hands	
Elbow/Forearm	
Knees	
Hips/Thighs	
Ankles/Feet	

* 15. Throughout your career as a podiatrist, have you ever had any work related musculoskeletal pain or injuries?

- ◯ Yes
- ◯ No

16. Please indicate the body region of your most significant pain occurrence or injury you have had as a result of your work as a podiatrist?

- ◯ Low Back
- ◯ Neck
- ◯ Upper Back
- ◯ Thumbs
- ◯ Shoulders
- ◯ Forearm/Wrist
- ◯ Knees
- ◯ Hip/Thigh
- ◯ Ankle/Feet
- ◯ Other (please specify)

17. At what age did your most significant pain occurrence or injury occur?

- ◯ Under 25
- ◯ 25 to 29
- ◯ 30 to 34
- ◯ 35 to 39
- ◯ 40 to 44
- ◯ 45 to 49
- ◯ 50 to 54
- ◯ 55 to 59
- ◯ 60 to 64
- ◯ 65 to 69
- ◯ 70 to 74
- ◯ 75 to 79
- ◯ 80+

18. At what stage in your career did you most significant pain occurrence or injury occur?

- ◯ During training as a podiatrist
- ◯ In the first 5 years after graduating
- ◯ 6 to 10 years after graduating
- ◯ 11 to 15 years after graduating
- ◯ 15+ years after graduating

19. Did this pain occurrence or injury lead you to change the way you practice as a clinician?

- ◯ Yes
- ◯ No

Comment

20. Did you report this pain occurrence or injury to you supervisor or manager?

- ◯ Yes
- ◯ No

Comment

* 21. We are now interested in very recent pain experiences. Please do not include pain that is a result of feverish illness or menstruation. In the last 4 weeks have you had any pain in your low back (in the area shown in the image below)?

- ◯ Yes
- ◯ No

22. If yes, was the pain bad enough to limit your usual activities or change your daily routine for more than one day?

- ◯ Yes
- ◯ No

23. If you had pain in your low back in the last 4 weeks, how often did you have the pain?

- ◯ On some days
- ◯ On most days
- ◯ Every day

24. If you had low back pain in the last 4 weeks, how long was it since you had a whole month without any low back pain (please select one option only)?

- ◯ Less than 3 months
- ◯ 3 months or more but less than 7 months
- ◯ 7 months or more but less than 3 years
- ◯ 3 years or more

25. If you had low back pain in the last 4 weeks, please indicate what was the usual intensity of your pain on a scale of 0 to 10, where 0 means "no pain" and 10 means the "worst pain imaginable" (please select only one response).

- ◯ 0
- ◯ 1
- ◯ 2
- ◯ 3
- ◯ 4
- ◯ 5
- ◯ 6
- ◯ 7
- ◯ 8
- ◯ 9
- ◯ 10

* 26. Have you had pain that goes down the leg?

- ◯ Yes
- ◯ No

27. If yes, has this pain spread below the knee?

- ◯ Yes
- ◯ No

* 28. Please indicate which job risk factors you feel may contribute to low back pain or injuries for podiatrists.

Rank your top 5 factors with 1 being the MOST risky and 5 LEAST risky by putting a number against the task.

Performing the same task over and over	
Working in the same position for long periods	
Treating a large number of patients in a single day	
Bending or twisting your back in an awkward way	
Lifting or transferring dependent patients	
Continuing to work when injured or hurt	
Reaching or working away from your body	
Working in awkward or cramped conditions	
Not enough rest breaks during the day	
Unanticipated sudden movement or fall by patient	
Assisting patient during gait activities	
Carrying, lifting or moving heavy materials/equipment	
Working with confused or agitated patients	
Inadequate training in injury prevention	

* 29. Have you ever had a significant musculoskeletal pain occurrence or injury that occurred through everyday living or sport that has impacted on your career?

- ◯ Yes
- ◯ No

If yes please specify

[ ]

30. If you have any comments about the questions or your responses within this survey, please add within the text box below.

31. Thank you for your time. If you wish to have the summary of the research results emailed to you at the end of the project, please enter your email below.
